# Abiotic Stress Signaling in Wheat – An Inclusive Overview of Hormonal Interactions During Abiotic Stress Responses in Wheat

**DOI:** 10.3389/fpls.2018.00734

**Published:** 2018-06-11

**Authors:** Kumar Abhinandan, Logan Skori, Matija Stanic, Neil M. N. Hickerson, Muhammad Jamshed, Marcus A. Samuel

**Affiliations:** Department of Biological Sciences, University of Calgary, Calgary, AB, Canada

**Keywords:** abiotic stress, signaling, heat shock factors, phytohormones, abscisic acid

## Abstract

Rapid global warming directly impacts agricultural productivity and poses a major challenge to the present-day agriculture. Recent climate change models predict severe losses in crop production worldwide due to the changing environment, and in wheat, this can be as large as 42 Mt/°C rise in temperature. Although wheat occupies the largest total harvested area (38.8%) among the cereals including rice and maize, its total productivity remains the lowest. The major production losses in wheat are caused more by abiotic stresses such as drought, salinity, and high temperature than by biotic insults. Thus, understanding the effects of these stresses becomes indispensable for wheat improvement programs which have depended mainly on the genetic variations present in the wheat genome through conventional breeding. Notably, recent biotechnological breakthroughs in the understanding of gene functions and access to whole genome sequences have opened new avenues for crop improvement. Despite the availability of such resources in wheat, progress is still limited to the understanding of the stress signaling mechanisms using model plants such as *Arabidopsis*, rice and *Brachypodium* and not directly using wheat as the model organism. This review presents an inclusive overview of the phenotypic and physiological changes in wheat due to various abiotic stresses followed by the current state of knowledge on the identified mechanisms of perception and signal transduction in wheat. Specifically, this review provides an in-depth analysis of different hormonal interactions and signaling observed during abiotic stress signaling in wheat.

## Global Food Security and the Present Status of Wheat Production

The crisis to feed the ever-growing population is compounded by the counteracting issues of spatial allocation of land for accommodation vs. agriculture. This issue of food insecurity is further amplified by degrading soil fertility conditions, reduced crop productivity and unpredictable climate change, which are expected to worsen in the near future. Although several policies addressing food security have been initiated (Carraro et al., 2015; FAO, 2015)^1^, one of the most challenging propositions is to achieve higher crop productivity under stressful environmental conditions. Agriculture as an occupation depends on the ability to cultivate crops suitable for a particular climate in a defined region. Prolonged exposure to high temperatures in rainfed areas of the world, may lead to drought stress. Exposure to high temperatures may also induce osmotic stress if water evaporates from soils resulting in elevated salt concentrations. Although drought and salt stress are the major stressors affecting crop production worldwide, the presence of a combination of these as well as heat is not uncommon and could lead to a drastic reduction in crop fitness and productivity.

**FIGURE 1:**
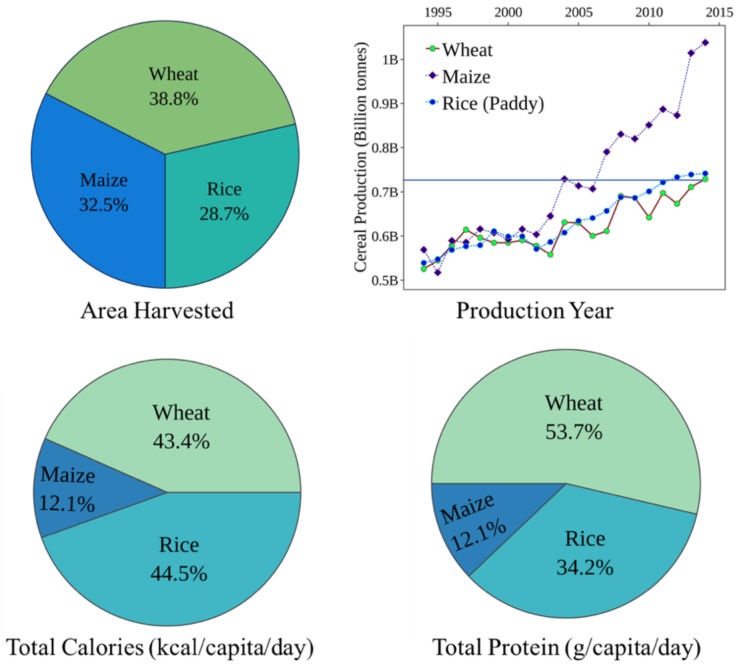
Production and consumption statistics for three global cereal crops: wheat, maize, and rice. Production statistics highlight the relative area harvested and yield production represented by billion tonnes/year. Consumption values highlight the total calories and protein per capita per day obtained by the three major cereal crops (FAO, 2015).

Among the main staple crops across the globe, cereals such as wheat, rice and maize are the most important for providing daily calories and protein intake. Of these, wheat was the first crop to be domesticated and forms the major staple food globally (Tack et al., 2015). Wheat tops the total harvested area (38.8%) and supplies significantly more protein per gram (12–15%) compared to rice or maize (2–3%) and thus serves as a better cereal of choice (**Figure 1**). Despite its sizeable cultivated area worldwide, the production levels are significantly lower than rice and maize (FAO, 2017). A meta-analysis of 1,700 published simulations (Challinor et al., 2014) predicted a significant yield loss in wheat in temperate and tropical regions with every 2°C rise. Similar climate modeling research predicted a 6% decrease in wheat production which is equivalent to a possible reduction of 42 Mt/°C (Asseng et al., 2014). Thus, maintaining crop productivity levels is a significant challenge in today’s agriculture, and the mitigation strategies must streamline toward boosting yield under limited resources. Conventional breeding to improve stress tolerance is time and labor intensive and involves multigene families that govern the molecular and physiological mechanisms. Interestingly, this mechanistic complexity is further magnified due to the striking differences in the tolerance levels of different cultivars under similar stress conditions.

One of the key principles of crop improvement involves utilizing the genetic variation in the gene pool and identifying the desired traits important for attaining stress tolerance. Bread wheat (*Triticum aestivum*) is a hexaploid, with an estimated ∼ 17 Gbp genome size that is composed of three closely related, but, independently maintained genomes: *Triticum urartu* (the A-genome donor), *Aegilops speltoides* (the B-genome donor) and *Aegilops tauschii* (the D-genome donor), formed as a result of a series of naturally occurring hybridization events. The natural genetic variation in the germplasm has helped breeders introgress new traits but has attained limited success due to the redundancy in the genomes (Brenchley et al., 2012; Panchy et al., 2016). However, recent biotechnological breakthroughs in the understanding of gene functions and the access to whole genome sequences have unearthed new avenues for crop improvement. Interestingly, despite the availability of such resources in wheat, the progress is still limited to the understanding of the stress signaling mechanisms using model plants such as *Arabidopsis*, rice (monocot) and *Brachypodium* (as a model plant for wheat). Considerable research and development in employing these biotechnological breakthroughs have lacked thus far in wheat improvement. This review focuses primarily on the hormonal interactions of the major abiotic stressors in wheat growth and development such as drought, salinity and high and low temperatures. However, other abiotic stresses such as UV-B, ozone and metal toxicity have also been shown to negatively affect crop growth. Reviews on the recent developments in the physiological responses to these other abiotic stressors in crops can be found elsewhere (Sharma et al., 2017 for UV-B, Rizwan et al., 2016 for Cadmium toxicity, and Mills et al., 2018 and Ainsworth, 2017 for the effects of ozone on crop production).

## Phenotypic and Physiological Changes to Abiotic Stresses in Wheat

### Osmotic Stress: A Major Convergence Point for Various Abiotic Stresses

Occurrences of osmotic stresses have profound impacts on global wheat production (Daryanto et al., 2016; Oyiga et al., 2016). Drought is a global problem, occurring in virtually any wheat producing region that can cause severe osmotic stress. Estimates indicate that drought stress in the United States is responsible for $6–8 billion per year in losses and increases the strain on global food security (Dai, 2011; Fontaine et al., 2014). Instances of drought are becoming more frequent and increasingly persistent due to global warming, elevating the potential to threaten yields (Dai, 2011). Soil salinization is another source of severe osmotic stress that threatens approximately 20% of arable farmland (Shrivastava and Kumar, 2015). Rates of soil salinization are rapidly increasing and are expected to affect 50% of arable cropland by 2050 (Ashraf, 2009; Shrivastava and Kumar, 2015). With the current technologies, increasing wheat production to preserve food security would mean that more acres of wheat will have to be grown on sub-marginal lands which are subjected to increasing instances of osmotic stress.

The exposure of wheat crops to osmotic stress can occur across all stages of plant development leading to cellular damage. Intensity and duration of osmotic stressors can influence the extent of cellular damage induced during wheat development (Sarto et al., 2017) that can influence growth and developmental processes, in most cases leading to compromised yield (Wang et al., 2003). The timing of osmotic stress exposure is critical as certain developmental stages appear to be more sensitive to osmotic damage (Dolferus et al., 2011; Mickky and Aldesuquy, 2017). Germination is an extremely sensitive stage as it influences crop density and uniform maturation, ultimately playing an important role in yield. Severe fluctuations in the immediate environment of a germinating seed can delay or inhibit germination processes, leading to potential yield loss due to reduced cropping density (Hampson and Simpson, 1990; Almansouri et al., 2001). Soil environments with elevated salt concentration or lacking water may hinder the ability of seeds to uptake moisture, which is required for proper germination (Dodd and Donovan, 1999). Germination delays may leave crops susceptible to temperature stress at the end of growing seasons or promote uneven maturation of crops (Briggs and Ayten-Fisu, 1979; Wardlaw et al., 1989; Rauf et al., 2007).

When drought/osmotic stress occurred in the later stages of tillering, yield was reduced as kernel number and plant recovery were reduced (Blum et al., 1989, 1994). Many other abiotic stress-related plant parameters such as leaf area index, dry matter accumulation, and net assimilation rates are also negatively affected by drought (Ihsan et al., 2016). Drought can disrupt metabolic processes including photosynthesis which can impair sugar synthesis required to drive yield in wheat crops (Shangguan et al., 1999; Subrahmanyam et al., 2006). Wheat subjected to drought during tillering also has profound effects on yield (Blum et al., 1989). The overlap of osmotic stress during anthesis and grain-filling can cause substantial yield loss by altering a suite of developmental processes and drought stress before or during anthesis has been shown to negatively impact grain number per plant (Liu H. et al., 2015; Dong et al., 2017). This reduction in grain number due to water stress can be caused by a multitude of reasons including: disrupted meristem development, floret abortion, and pollen sterility (Ji et al., 2010; Dolferus et al., 2011; Dong et al., 2017). Drought susceptible wheat cultivars are known to have higher canopy temperature and incurred more yield loss under water stress (Blum et al., 1989).

It has also been suggested that male fertility is impacted by endogenous ABA levels within the anther tissue (Ji et al., 2010). Cultivars that are less sensitive to water stress had lower levels of anther ABA and subsequently improved grain number (Ji et al., 2010; Dong et al., 2017). Following anthesis, drought stress can inflict a more profound impact on grain filling leading to a shortened period of grain filling and altered enzymatic activities (Ahmadi and Baker, 2001; Shah and Paulsen, 2003; Pireivatlou, 2010; Farooq et al., 2014). However, when established, terminal priming of wheat plants to low soil relative water content (20–40%) resulted in higher grain yield than the non-primed plants indicating that plants acclimate to stress by the production of metabolites essential for stress tolerance and recovery (Wang et al., 2014, 2015).

Salt stress induces similar effects on wheat when compared to drought (Grieve et al., 1993; Francois et al., 1994). Salinity hinders leaf growth and tillering in stressed plants by halting the leaf primordia initiation rates without affecting the developmental stages, leading to leaf number, size and number of tillers (Grieve et al., 1993, 1994). Salt stress occurring before terminal spikelet development led to reduction of various yield parameters including kernel weight and number (Francois et al., 1994). When salt stress was applied after terminal spikelet development, there was a reduction in kernel number and weight (Francois et al., 1994). It is also important to note the importance of the flag leaf when looking at yield and grain filling (Abbad et al., 2004; Guóth et al., 2009; Biswal and Kohli, 2013; Borrill et al., 2015). The flag leaf contributes approximately 30–50% of seed carbohydrates; therefore, any damage induced to the flag leaf would negatively impact yield (Sylvester-Bradley et al., 1990; Farooq et al., 2014). Osmotic stress induced by drought and ion toxicity can accelerate flag leaf senescence, which reduces chlorophyll content leading to reduced photosynthetic activity (Zheng et al., 2008). At the cellular level, high salinity disrupts selective ion absorption due to low water content and thus affects nutrient availability (Davenport and Tester, 2000). Differences in saline tolerance in cultivars are caused by the differential selectivity of K^+^ over Na^+^ and lower rates of Na^+^ accumulation in the above-ground tissues of the plants (Apse and Blumwald, 2011; Nieves-Cordones et al., 2014). Therefore, ionic discrimination between K^+^and Na^+^is the key to survivability under high salt stress (Kronzucker and Britto, 2011).

Similar to drought stress, high salinity also induces high levels of reactive oxygen species (ROS) that damage various cellular functions (Apel and Hirt, 2004). Notably, increased salinity affects membrane permeability in *Hordeum vulgare*, one of the most tolerant cereal crops (Mansour and Stadelmann, 1994). Other physiological parameters such as gas-exchange (stomatal closure) and photosynthetic rates are also affected by osmotic stress and have been shown to decrease due to oxidative damage induced by osmotic stress (Farooq et al., 2014; Ihsan et al., 2016). The effects of nitrogenous fertilizers were directly correlated to the water status of the soil and transpiration rates of wheat plants (Tanguilig et al., 1987; Bloem et al., 1992). The susceptible cultivars of wheat were shown to have decreased cytoplasmic viscosity, whereas, tolerant varieties maintained higher viscosity under high salt concentration. Thus, characteristics such as membrane permeability and cytoplasmic viscosity are important in deciding salt tolerance and vary widely among crop species (Mansour and Stadelmann, 1994). The physiological and phenotypic responses that occur in wheat plants is highly dependent on the ability to perceive and induce downstream signaling components.

However, osmotic responses vary for wheat cultivars with different ploidy levels. A meta-analysis performed on ∼ 300 reports based on tolerance levels of *2n*, *4n*, and *6n* cultivars revealed that hexaploid cultivars were more tolerant to drought stress than the diploid and tetraploid cultivars (Wang et al., 2017). Interestingly, this is contradictory to the previous report by Zhang and Kirkham (1994), which claimed higher susceptibility of hexaploid wheat because of less efficient antioxidant systems than the diploid and tetraploid wheat. Such differences in observations can arise from the variability in cultivars used as the acclimation to drought stress is usually attributed to the ability to induce antioxidant defense and repair mechanisms (Rampino et al., 2006; Khanna-Chopra and Selote, 2007).

### Temperature Stress

Temperature plays an important role regulating normal crop growth and development, ultimately determining yield (Porter and Gawith, 1999; Bita and Gerats, 2013; Kosová et al., 2013; Tardieu, 2013; Gray and Brady, 2016). Wheat is grown globally due largely in part to the broad temperature range which wheat can withstand. Survival of wheat occurs between the upper and lower limits of temperature lethality which is roughly 47.5 ± 0.5°C and -17 ± 1.2°C, respectively (Porter and Gawith, 1999). Approaching either temperature threshold will have detrimental effects on plant growth and development (Porter and Gawith, 1999; Thakur et al., 2010; Farooq et al., 2011). Like osmotic stress, the effect of temperature stress on wheat production is heavily reliant on the wheat development stage that is subjected to temperature stress (Porter and Gawith, 1999). Germination of wheat is susceptible to temperature stress and seeds experience delays in germination due to altered metabolic activity because of surrounding soil temperatures (Jame and Cutforth, 2004). Delay in germination and emergence can alter plant density and early crop establishment leading to a prevalence of high temperatures during anthesis and seed set leading to significant yield loss (Wardlaw et al., 1989; Hampson and Simpson, 1990; Almansouri et al., 2001). Thus, temperature is a significant variable determining farming practices such as time of planting as well as harvesting and therefore, fluctuations in temperature during the growing season can cause severe crop losses (Ottman et al., 2012; Iizumi and Ramankutty, 2015; Yang Z. et al., 2017).

Cold stress is particularly challenging for winter-grown wheat crops grown in temperate and arid regions (Thakur et al., 2010). Exposure to cold temperatures alters various biochemical processes including photosynthesis (Wardlaw et al., 1989; Thakur et al., 2010; Li et al., 2014) and can induce membrane damage, contributing to reduced plant performance (Steponkus, 1984). Crop hardening is particularly important in winter grown wheat crops, where wheat seedlings gradually acclimate to cool conditions. Priming of crops helps to alleviate damage and improve stress tolerance through various mechanisms including photosynthetic apparatus preservation (Li et al., 2014). During the reproductive phase, wheat is particularly sensitive to cold stress (see Thakur et al., 2010 for a detailed review) where cold temperatures can affect grain number if stress occurs prior to anthesis (Dolferus et al., 2011). Cold temperatures can disrupt gametophyte tissue development, specifically in pollen tapetal cells leading to pollen sterility (Subedi et al., 1998a,b; Ji et al., 2017); pollen tube elongation is also disrupted by low-temperature stress (Chakrabarti et al., 2011). The effect of low temperature stress on grain filling is due to altered sink-source distribution resulting in reduced nutritional reserves being diverted to the developing seed (Hunt et al., 1991; Subedi et al., 1998a; Thakur et al., 2010).

High-temperature stress is also problematic in wheat following seedling emergence (see Farooq et al., 2011 for a detailed review). Exposure of wheat to gradually increasing temperature will allow for a priming phase that will improve plant performance due to increased stress tolerance (Wang et al., 2011). Since anthesis and panicle emergence are considered to be the most prone stages to temperature stress, pre-anthesis priming of wheat plants showed less severe post-anthesis damage (Wang et al., 2011). High temperature can directly affect photosynthesis due to photosystem damage and enzyme impairment leading to reductions in yield (Xu et al., 1995; Al-Khatib and Paulsen, 1999; Wahid et al., 2007; Farooq et al., 2011), affecting pollen quality and reduce seed set in wheat (Hays et al., 2007). High temperature has the potential to disrupt pollen development prior to anthesis, contributing to reduced seed set (Ferris et al., 1998; Farooq et al., 2011). Photosynthetic perturbations caused by high temperature can also induce early senescence which can reduce assimilates required for proper grain filling (Harding et al., 1990; Machado and Paulsen, 2001). Accelerated senescence and death of wheat plants were observed under greenhouse conditions when plants were exposed to continuous high temperature of 30–40°C irrespective of the imposed drought (Machado and Paulsen, 2001). Furthermore, a decrease in dry matter was also observed under high temperature conditions providing a direct correlation to reduced growth (Ihsan et al., 2016). Exposure of wheat crops to high-temperature during reproductive phase can negatively impact grain number and grain filling (Blum et al., 1994; Corbellini et al., 1997; Ferris et al., 1998; Dias and Lidon, 2009; Chakrabarti et al., 2011; Farooq et al., 2011). Seed size can be influenced by high temperatures during kernel filling stage, due to higher respiration rates and affects many other qualitative losses in the flour quality along with 3 – 4% loss in yield/°C rise over 15°C (Wardlaw et al., 1989; Hays et al., 2007).

## Overview of Abiotic Signaling Pathways

### Osmotic Stress Perception and Transduction

The first event in the stress response pathway of an organism is the detection or perception of stress. Any alterations in osmotic conditions in the cell (induced by salt-stress, temperature, or water availability) can induce physical alterations in the plasma membrane (Willing and Leopold, 1983). Plant cells contain integral or peripheral mechanosensors on their membranes allowing them to detect such mechanical changes induced by stresses. Plant homologs of such sensors in bacterial systems have been found and include the *Msc-like* and *Piezo* homologs which have been linked to survivability under dehydration stress in plants (Zhu, 2016). Also, it is known that mechanical perturbations perceived by plant cells play critical roles in developmental processes such as phyllotaxis (Sampathkumar et al., 2014; Louveaux et al., 2016). One of the most common responses to abiotic stresses is the increase in free cytosolic calcium levels (Knight and Knight, 2001), suggesting possible roles of ion channels as mechanosensors (Monshausen and Haswell, 2013). A plasma-membrane protein OSCA1, capable of functioning as a hyperosmolarity-gated ion channel was identified in *Arabidopsis*, and is thought to be responsible for the initial free Ca^2+^ spike upon detection of osmotic stress (Yuan et al., 2014). Another putative osmosensor discovered in *Arabidopsis* is AtHK1, a transmembrane histidine kinase (Urao et al., 1999) which could complement the osmosensitive yeast double mutants that lacked two osmosensors *sln1/sho1* (Urao et al., 1999). The involvement of receptors other than OSCA1 are yet to be identified; it is clear that relative levels of toxic ions in the osmotic pool would differentially trigger activation of signaling pathways which would allow the cells to regulate ion concentration inside the cytoplasm selectively. Interestingly, plants have evolved mechanisms which keep sodium (toxic ion) levels within tissues low via compartmentalization into the vacuole (Ji et al., 2013).

High salinity can trigger two distinct stresses: (1) High salt levels in the soil can cause a decrease in soil water potential which makes it difficult for plant roots to uptake water, leading to a physiological drought condition. (2) Sodium accumulation in tissues can reach toxic levels and can cause ionic stress. As sodium is taken up through ion transporters or anatomical leaks in the root endodermis, it disrupts normal cellular metabolism (Davenport and Tester, 2000; Tester and Davenport, 2003). Osmotic stress signaling research has primarily been conducted in *Arabidopsis*, which has served as a foundation for understanding stress signaling in wheat. ABA-dependent and ABA-independent pathways are known to mediate signal transduction during osmotic stress (Yamaguchi-Shinozaki and Shinozaki, 2005; Agarwal and Jha, 2010). Perception and signal transduction of osmotic stress also depends on secondary messengers (Ca^2+^, ROS, nitric oxide), which play essential roles in physiological responses such as stomatal closure (Roychoudhury et al., 2013). The immediate signals of abiotic stresses are transduced through increased levels of ROS, such as singlet oxygen (^1^O_2_), superoxide anion radical (O_2_^⋅-^), hydroxyl radical (HO^⋅^) and hydrogen peroxide (H_2_O_2_) (Reczek and Chandel, 2015).

The ABA-mediated pathway is discussed in later sections in this review; the ABA-independent pathway is mediated by multiple families of regulatory transcription factors (TFs) including the dehydration-responsive element-binding (*DREB*) protein/C-repeat binding factors (CBFs) family. Osmotic stress can signal the induction of DREB1/CBF members, which can interact with DRE/CRT motifs to induce downstream expression of stress-induced genes, independent of ABA (Maruyama et al., 2004). ABA-independent DREB2 is crucial to osmotic stress signaling and can induce gene expression by interacting with DRE/CRT promoter elements (Maruyama et al., 2004). In addition to DREBs, NAC domain proteins can regulate expression of stress-inducible genes independent of ABA (Tran et al., 2007). Zinc finger homeodomain (ZFHD) proteins can also regulate stress-responsive genes independent of ABA except for ZFHD1, which was responsive to ABA treatment (Tran et al., 2007).

Ionic stress in plants has been shown to be mediated via the Salt Overly Sensitive (SOS) pathway involving calcium signaling (Ji et al., 2013). High sodium concentrations cause induction of a cytosolic calcium signal which then initiates the SOS pathway (Halfter et al., 2000). SOS3, a calcium-binding protein, detects the initial salt-stress-induced calcium spike and activates SOS2, a serine/threonine protein kinase (Quan et al., 2007). SOS2 is then responsible for activating the Na^+^/H^+^ antiporter SOS1 which mediates the return to ionic homeostasis (Ji et al., 2013). Another pathway which leads to the activation of the SOS1 antiporter is the Phospholipase D pathway (Bargmann and Munnik, 2006). Upon detection of salt-stress, phospholipase D alpha 1 activity spikes causing an accumulation of its catalytic product phosphatidic acid (PA). PA, in turn, activates MPK6, which then phosphorylates/activates SOS1, resulting in sodium trafficking out of the cell/tissue (Yu et al., 2010). An essential pathway for mediating salt stress in yeast is the HOG MAPK pathway responsible for the reorganization of the actin cytoskeleton (Saito and Posas, 2012). In yeast, under hyperosmotic stress, HOG1 activates NHA1 and TOK1 ion transporters and migrates to the nucleus to activate stress-related TFs which induce glycerol biosynthesis genes which assist in the return of the system to osmotic homeostasis (Proft and Struhl, 2004). Plants may utilize a homologous mechanism as it is known that salt-stress induces the reorganization of microtubules in plant cells (Brewster and Gustin, 1994).

### Heat Perception and Signal Transduction

At the cellular level, high temperature can lead to misfolding, denaturation or loss of function of proteins which affects optimal cellular functions triggering stress responses. Temperature fluctuation-induced changes in plasma membrane fluidity have been associated with the generation of PA and phosphatidylinositol 4,5-bisphosphate (PIP2) (Mishkind et al., 2009). Whether PA, PIP2 or related molecules have any role in perception is unclear, but, it was shown that PIP2 is generated through activation of PIPK, and PIP2 requires small monomeric G-proteins or the α-subunit of the heterotrimeric G-protein for its activation (Mishkind et al., 2009). Such receptors which respond to elevated temperatures are yet to be characterized, and the role of self-activating heterotrimeric G-protein (Urano et al., 2013) is still under investigation. Temperature fluctuations can also trigger responses in the nucleus where an alternative form of histone (H2A.Z) is known to directly regulate the unwrapping and occupancy of heat regulated gene expression under elevated temperature in *Arabidopsis*, and this action of H2A.Z was independent of transcription (Kumar and Wigge, 2010). Heat stress may be perceived via chaperone-mediated signaling proteins, wherein, under non-stressed conditions, chaperone proteins may be bound to heat-stress response TFs (Heat shock factors; HSFs) keeping them inactive. Upon elevation of temperatures, the chaperone proteins dissociate from the heat-stress response transcription factors, and this allows them to bind to heat-stress responsive genes known as heat shock elements (HSE) and activate downstream physiological responses (Zhu, 2016). Under non-stressed conditions, bZIP28 (ER membrane bound TF) is bound by the chaperone BIP preventing its movement into the nucleus. Stress-induced protein unfolding or misfolding abolishes the BIP-bZIP28 interaction that mobilizes bZIP28 to the Golgi where it is proteolytically processed for its migration to the nucleus which activates transcriptional changes (Gao et al., 2008; Liu and Howell, 2016).

**FIGURE 2:**
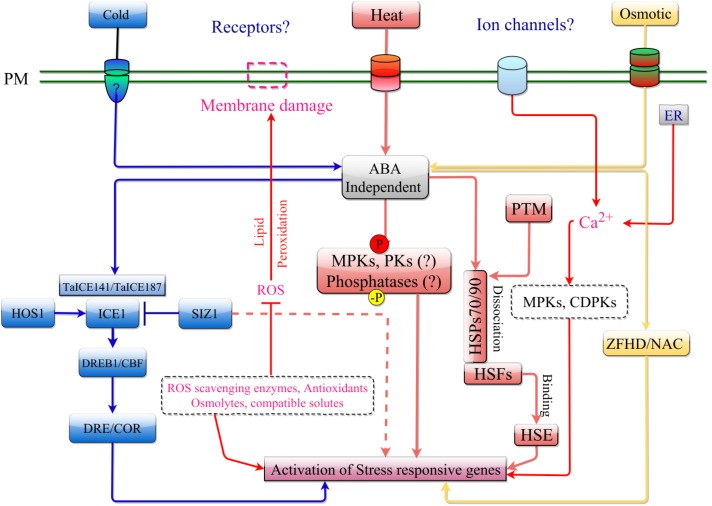
ABA-independent signaling pathway in response to cold, heat, and osmotic stress. The stress is perceived by receptor/ion channel such as OSCA1 and COLD1 present on the plasma membrane. ABA-independent signaling in response to cold stress is mediated by the ICE1 signaling cascade involving DREB. Osmotic stress is transduced in a similar manner as cold stress but can activate stress response by regulating ZFHD and NAC transcription factors. Heat stress can activate stress response through induction of ROS. This leads to activation of MPKs, protein kinases (PKs) and other phosphatases, that can trigger the Heat Shock Factors (HSF), which is kept from entering the nucleus by HSPs (HSP70/90 complex). An increase in misfolded cytosolic proteins following heat shock, can destabilize this complex allowing HSF to enter the nucleus and induce transcription of stress responsive genes by binding to their HSE.

Heat stress changes membrane fluidity which leads to rapid generation of ROS and increased Ca^2+^ influx (Los and Murata, 2004; Horváth et al., 2012; Kurusu et al., 2012). Like other abiotic stresses, ROS and Ca^2+^ can trigger heat shock responses by activating the heat shock TF, HsfA1, which is referred to as the master-regulator of heat responses in plants (Ohama et al., 2017). However, the cytoplasmic pool contains multiple forms of heat shock proteins (HSPs), the levels of which are significantly upregulated under heat stress conditions (Driedonks et al., 2015). The upregulation of these HSPs are controlled through the activation of HSFA1, the activation of which is proposed to occur in two possible pathways, the chaperone titration model (Richter et al., 2010; Ohama et al., 2016) and HsfA1-independent heat shock response (Sangwan et al., 2002; Volkov et al., 2006; Ohama et al., 2016). In chaperone titration model, HSP70 and HSP90 are shown to bind HsfA1, which prevents activation of HsfA1 by another regulatory protein involved in post-translational modifications of HsfA1 (Hahn et al., 2011). During heat stress, the stoichiometric increase in the abundance of unfolded proteins leads to the destabilization of the HSFA1-HSP70-HSP90 complex, ultimately releasing the HsfA1 for its entry into nucleus and inducing the transcription of heat shock response genes (Hahn et al., 2011) (**Figure 2**).

The HsfA1-independent activation occurs through an increase in response to oxidative load and free cytosolic Ca^2+^ (Zheng et al., 2012), where the increase in free Ca^2+^ levels is correlated with IP3 levels leading to activation of DREB2A (Zheng et al., 2012). The activation of DREB2A has also been observed during osmotic stress signaling, indicating that activation of HSFs is not limited to the regulation of HSPs, but, otherwise forms an elaborate network that regulates a multitude of functions (Ohama et al., 2017). Compared to non-plant species such as yeast and humans which only have 1 and 3 HSFs respectively, *Arabidopsis*, rice, and wheat have 21, 23, and 56 HSFs respectively (Scharf et al., 2012; Xue et al., 2014). The increased numbers of these HSFs indicate that these genes are important for diverse cellular functions and likely became increasingly redundant to overlap or complement metabolic needs (Dubcovsky and Dvorak, 2007). Interestingly, out of 56 known HSFs in wheat, only a few have been characterized for their roles in signaling. Wheat TaHsfA4a was shown to confer resistance to heavy metal toxicity (Cd) in yeast strains, and overexpression of *TaHsfA4a* in rice plants led to Cd tolerant plants (Shim et al., 2009), whereas the overexpression of *TaHSF3* (HsfB2a) in *Arabidopsis* resulted in thermo-tolerant plants (Zhang et al., 2013). Similarly, overexpression of *TaHsfA6f* in wheat led to heat stress protection by the transactivation of many small HSPs (TaHSP16.8, TaHSP17, TaHSP17.3), and TaHSP90.1-A1 (Xue et al., 2015). In another finding, overexpression of *TaHsfC2a-B*was shown to induce expression of *TaHSP70d* and *TaGalSyn* conferring tolerance to heat stress in an ABA-dependent manner (Hu et al., 2017), and *TaHSP26* overexpression was shown to confer tolerance of *Arabidopsis* to continuous exposure to high heat (Chauhan et al., 2012). Other TFs such as *TaWRKY1*, *TaWRKY33*, and *TaNAC2L* offered significant tolerance to heat stress when overexpressed in *Arabidopsis* (Guo et al., 2015; He et al., 2016). A simplified signal transduction pathway has been shown in **Figure 2**.

### Cold Perception and Signal Transduction

In contrast to sensing of elevated temperatures, there has been limited success in identifying receptors involved in the perception of the low-temperature stress signal. A recent genetic and biochemical analysis of CHILLING- TOLERANCE DIVERGENCE 1 (COLD1) protein indicates that it could act as a sensor for low temperature (Ma Y. et al., 2015). COLD1 is membrane (PM and ER)- localized and knocking down of *COLD1* in rice led to reduced free cytosolic Ca^2+^ (Ma Y. et al., 2015). COLD1 interacts with rice G-protein α-subunit 1 (RGA1) and functions as a GTPase accelerating factor/protein (GAP) leading to downstream signaling mediated through Ca^2+^, although the role of calcium under chilling stress is still elusive. Thus, it would be safe to theorize that the temperature gradients in other cereals might trigger similar kinds of proteins as that of COLD1 or other heterotrimeric G-proteins, diverged to identify subtle changes in the environment, but not limited to temperature. For example, a recent finding shows that the activation of HSFs, described earlier, is also achieved by osmotic and ROS fluctuations and were selectively regulated by the upstream mitogen-activated protein kinase (MAPK) signaling cascade (Perez-Salamo et al., 2014). Therefore, it is reasonable to assume that multiple stress signals could converge on the same intracellular signaling components to effect a response.

Cold stress can be characterized into two primary groups: (1) chilling stress, where temperatures remain above the freezing point but remain under optimal growing temperatures and (2) freezing stress, where temperatures drop below the freezing point. Cold stress signaling research has predominantly been conducted in the model organism *Arabidopsis*, and recent advancements have identified parallel processes in species such as wheat (Shen et al., 2003; Egawa et al., 2006; Badawi et al., 2008). The global response to abiotic stress leads to the production of stress response regulatory proteins (TFs) or the downstream production of protective proteins or metabolites (Ahuja et al., 2010). Perception of cold stress leads to a variety of responses which include antioxidant production, ROS production, Ca^2+^ release, and activation of multiple transcriptional cascades (Knight and Knight, 2001; Agarwal et al., 2005a,b). Cold signaling is also segregated into two major signaling pathways: (1) ABA-independent and (2) ABA-dependent (Ishitani et al., 1997; Leung and Giraudat, 1998; Chinnusamy, 2003; Knox et al., 2008; Roychoudhury et al., 2013).

The ABA-independent signaling pathway responds to cold stress without the requirement of ABA activation. In *Arabidopsis*, cold stress has been shown to induce expression of *ICE1*, encoding a bHLH TF that is capable of activating downstream *DREB1/CBF* members (Chinnusamy, 2003). In wheat, homologs of *ICE1* have been identified as *TaICE141* and *TaICE187* which regulate *CBF* genes, demonstrating conservation of cold stress signaling between species (Badawi et al., 2008). Overexpression lines of *ICE1* and *TaICE141/TaICE187* in *Arabidopsis* subjected to cold stress demonstrated the importance of *ICE* homologs for enhanced cold tolerance (Badawi et al., 2008). Induced DREB1A/CBF3 can then interact with DRE/CRT promoter elements to regulate the expression of various cold-responsive genes (Liu et al., 1998; Novillo et al., 2004). Persistent ICE1 activity is known to be regulated by protein modifications; sumoylation of ICE1 by SIZ1 can increase the stability of ICE1, driving a prolonged cold response (Miura et al., 2007). The activation of various cold-responsive genes downstream can then mitigate the effects of cold stress. To moderate or repress the cold response, ICE1 can be polyubiquitinated by HOS1, which targets ICE1 for 26S proteasomal-mediated degradation (Dong et al., 2006).

**FIGURE 3:**
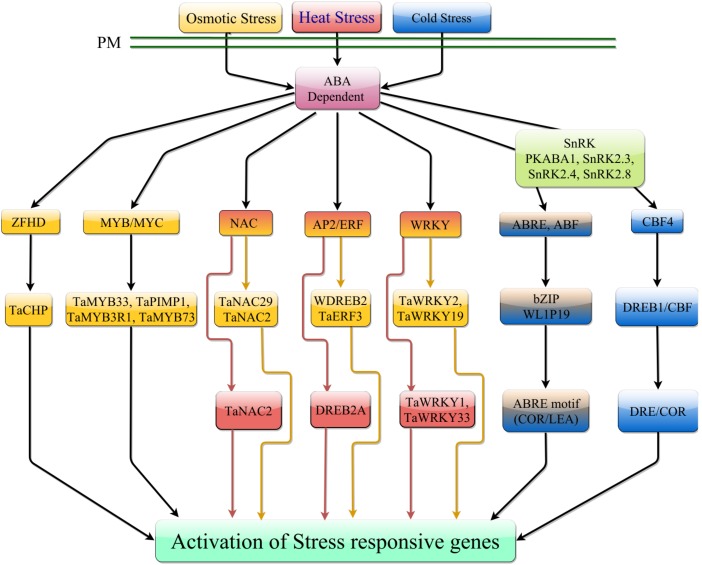
ABA-dependent signaling pathway in response to temperature stress. Under heat stress, ABA-dependent signaling is mediated by NAC, AP2/ERF, and WRKY TFs. Under cold stress, ABA can promote expression of *CBF4* which can mediate transcription of downstream stress responsive genes. ABA interaction with receptor components can activate phosphorylation of ABREs which can mediate downstream gene expression. In response to osmotic stress, ABA can induce transcription of numerous TF families, which induce expression of stress responsive genes. Osmotic stress can also induce activation of ABREs which can mediate expression of downstream stress responsive genes.

The ABA-dependent role in cold stress signal transduction is also required for normal response to cold stress (**Figure 3**). ABA-dependent signal transduction is discussed in later sections. Increasing ABA content in response to cold stress can facilitate the accumulation of various second messenger molecules including Ca^2+^ and ROS (Agarwal et al., 2005b). *Arabidopsis* FRY1, required for inositol triphosphate (IP3) turnover, functions as a negative regulator of ABA and abiotic stress responses and functions in attenuating these responses (Xiong et al., 2001). In wheat seedlings, ABA application induces a marked increase in Ca^2+^, which can subsequently increase the activity of NADPH oxidase leading to accumulation of hydrogen peroxide (Agarwal et al., 2005b). An increase in ABA and cytosolic Ca^2+^ can also signal the activation of calcium-dependent protein kinases (CDPKs) to mitigate the effects of various abiotic stresses. Li et al. (2008) analyzed 20 CDPKs in wheat and identified seven different CDPKs that responded to exogenous ABA treatment. From the ABA-responsive CDPKs, CDPK9 was responsive to drought and salinity suggesting that CDPK may play a role in abiotic response and integration of various stresses (Li et al., 2008). A simplified signal transduction pathway integrating cold and osmotic stress has been shown in **Figure 2**.

## Advancements in Hormone-Dependent Abiotic Stress Signaling

**FIGURE 4:**
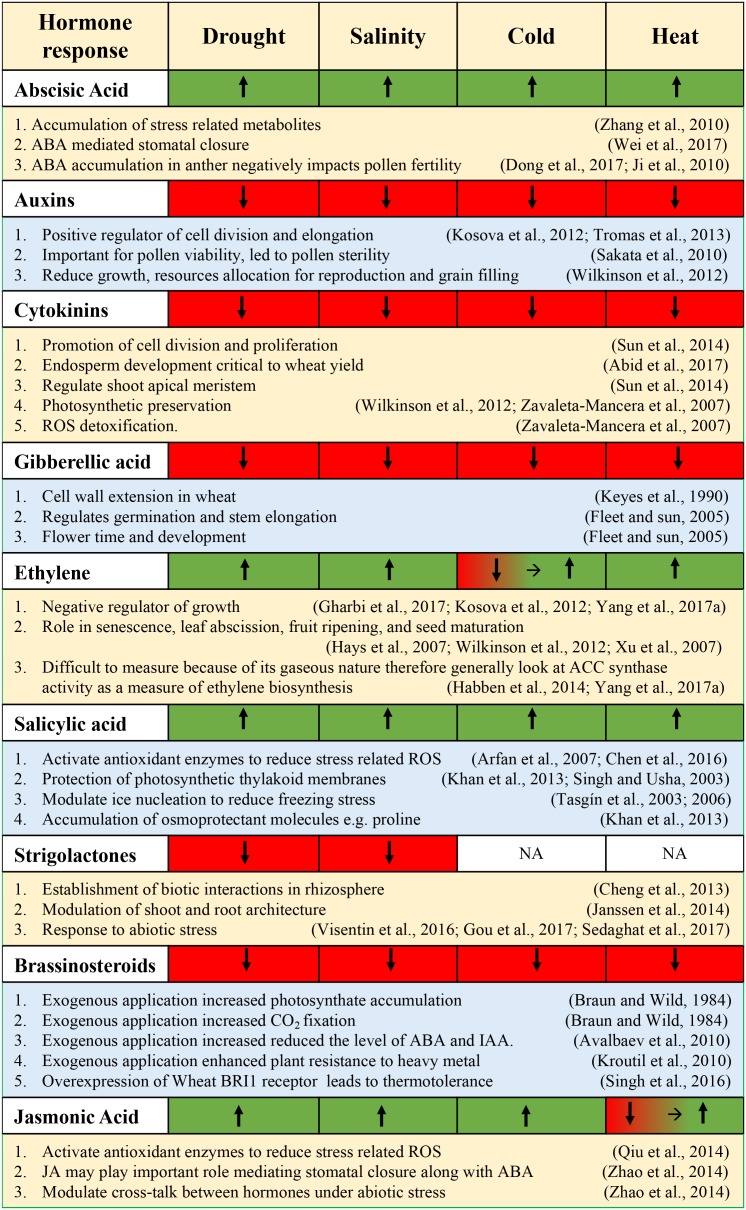
Summary of hormonal responses [upregulated (green), downregulated (red)] following exposure to various abiotic stresses.

This section will address recent advances in wheat abiotic stress responses that interact with the various phytohormones and highlight the characterization of essential regulatory proteins and TFs playing a role in connecting the abiotic stress signals to the hormonal network. Along with important TFs, this section will also comment on the essential downstream genes that are important for abiotic stress tolerance. The production of osmoprotectants and metabolic products induced by hormone-dependent signaling will also be addressed. Lastly, this section will discuss any agronomic improvements that are mediated by hormone-dependent pathways (e.g., nutrient supplementation). **Figure 4** summarizes the hormonal responses during various abiotic stresses.

### Abscisic Acid, a Central Regulator of Abiotic Stress Tolerance in Wheat

ABA-mediated signaling begins with ABA interaction forming a ternary complex with the PYR/PYL/RCAR receptors and PP2C (Ma Y. et al., 2009). This interaction allows phosphorylation of SnRK2 proteins which can then subsequently phosphorylate AREB/ARFs (Furihata et al., 2006). The role of regulatory protein kinases is essential for phosphorylation of downstream targets in ABA-dependent signaling, initiating a cascade of transcriptional events (Holappa and Walker-Simmons, 1995; Johnson et al., 2002; Furihata et al., 2006; Umezawa et al., 2009). The role of SnRKs have been characterized extensively in *Arabidopsis*, and recent research in wheat has led to the identification of other SnRK2 homologs that appear to play a role in ABA-mediated abiotic stress signaling. The isolation and characterization of *TaSnRK2.3, TaSnRK2.4*, and *TaSnRK2.*8 suggest that these are essential players in abiotic stress signal transduction (Mao et al., 2010; Zhang et al., 2010; Tian et al., 2013). The expression of these wheat SnRK homologs can be induced by NaCl, drought (PEG), cold stress, and exogenous ABA application (Mao et al., 2010; Zhang et al., 2010; Tian et al., 2013). Overexpression of *TaSnRK2.3* and *TaSnRK2.8* in *Arabidopsis* increased tolerance to drought, saline, and cold conditions which were attributed to the increased accumulation of stress-related metabolites such as proline and increased expression of ABA-dependent and independent stress-responsive genes (Zhang et al., 2010). Increased transcript abundance of ABA biosynthetic genes (*ABA1, ABA2*) and signaling components (*ABI3, ABI4, ABI5)* as well as ABA-independent genes (*CBF1, CBF2, CBF3)* were reported in *TaSnRK2.8* overexpression lines indicating that tolerance to stress is an intricate genetic and physiological mechanism (Zhang et al., 2010). Recent research isolated 10 *SnRK2* homologs in wheat and conducted expression analysis in response to osmotic and cold stress (Zhang H. et al., 2016). Based on expression data and motif analysis, *TaSnRKs* were categorized into three subclasses (Zhang et al., 2010). TaSnRK2 subclass III was strongly induced by exogenous ABA and was shown to interact with TaABI1 (PP2C) to regulate ABA responses (Zhang et al., 2010). TaSnRK2 subclass I and II respond to osmotic and cold stress, however, signaling proceeds in an ABA-independent manner (Zhang et al., 2010). Endogenous ABA levels are primarily regulated by upstream biosynthetic genes namely *TaNCED*, which encodes 9-*cis*-epoxycarotenoid dioxygenase responsible for a cleavage reaction producing xanthoxin (Tong et al., 2017). The expression of the wheat *NCED* homolog was responsive to osmotic stress and exogenous ABA application (Tong et al., 2017). When *TaNCED* was overexpressed in *Arabidopsis*, increased ABA and proline synthesis led to lower water loss due to reduced stomatal conductance (Tong et al., 2017). Although this led to enhanced drought tolerance, it resulted in delayed germination which could have significant impacts on yield development, as previously discussed (Tong et al., 2017).

Transcription factors play crucial roles in gene regulation, and many families of TFs have been characterized to regulate ABA-dependent responses to abiotic stress. These TFs primarily include the bZIP, dehydration-responsive element-binding factors (DBFs), MYB/MYC, NAC and WRKY-type family TFs, which will be explored in this section. A wheat bZIP protein, WLIP19, was induced by drought, cold, and exogenous ABA application (Kobayashi et al., 2008). WLIP19 induction also led to increased transcriptional levels of previously characterized *COR/LEA* genes *WDHN13, WRAB17, WRAB18, WRAB19* (Ohno et al., 2003; Egawa et al., 2006; Kobayashi et al., 2008). When *WLIP* was constitutively overexpressed in tobacco, there was increased tolerance to cold and drought stress, suggesting a conserved role in the regulation of abiotic response (Kobayashi et al., 2008).

The dehydration-responsive element-binding factors (DBFs) are classified under AP2/ERF family, and wheat *TaAIDFa* was shown to be responsive to drought, cold stress, and exogenous ABA (Xu et al., 2008). The overexpression of *TaAIDFa* in *Arabidopsis* increased the transcript accumulation of *RD29A, COR15A*, and *ERD10* leading to increased tolerance under osmotic and cold stress (Xu et al., 2008). These findings also suggest the intricate link between osmotic and cold stress signaling. *WDREB2*, another AP2/ERF family TF, is a homolog of *DREB2* protein and was induced in response to osmotic and cold stress and, to a lesser extent, to exogenous ABA application (Egawa et al., 2006). It was also shown that *WDREB2* induction led to the accumulation of *WRAB19* transcript suggesting it could be a direct downstream COR/LEA target (Egawa et al., 2006). Interestingly, alternative splicing of *WDREB2* occurred depending on the abiotic stress encountered, which suggested the potential for an ABA-independent pathway regulating cold stress and an ABA-dependent pathway regulating osmotic stress (Egawa et al., 2006). The characterization of *TaDREB1* has also provided valuable information regarding its possible activation in an ABA-dependent manner. *TaDREB1* was found to respond to cold and salt stress, along with ABA application (Shen et al., 2003). A winter wheat cultivar had increased cold tolerance and had elevated expression of *TaDREB1*, leading to downstream accumulation of *WCS120.* (Shen et al., 2003) Under salt stress and ABA application, *TaDREB1* expression was reduced and no *WCS120* transcript accumulated suggesting other regulatory mechanisms in response to these stresses (Shen et al., 2003).

Overexpression of *TaERF3* (an ethylene-response factor) in wheat resulted in increased tolerance to salt and drought stress (Rong et al., 2014). *TaERF3* overexpression also led to a reduction of H_2_O_2_ content and increased endogenous proline levels, suggesting multiple downstream targets (Rong et al., 2014). The promoter sequence of *TaERF3* contained both ACGT and ABRE motifs suggesting abiotic stress response is likely mediated through an ABA-dependent manner. The activation of TaERF3 drove the downstream expression of multiple stress-related genes containing GCC-motifs including *RAB18* and *LEA3* (Rong et al., 2014).

The ABA-dependent signaling pathway also depends on the MYB/MYC TF family (Abe et al., 2003). Characterization of 60 MYB genes in wheat led to the identification of 15 genes (nine induced, six repressed) regulated by ABA (Zhang L. et al., 2012). Of these, 14 also responded to osmotic stress caused by PEG or salinity treatment indicating a role for MYB family of proteins in the ABA-dependent abiotic stress signaling pathway (Zhang L. et al., 2012). Furthermore, *TaMYB32* and *TaMYB33* improved salt tolerance when overexpressed in *Arabidopsis* (Qin et al., 2012; Zhang L. et al., 2012). Interestingly, increased expression of P5C synthetase was also observed suggesting that proline content was elevated in overexpression lines (Qin et al., 2012). There also appeared to be upregulation of *ZAT12*, which acts as a master TF involved in antioxidant defense system (Davletova et al., 2005). Together, *TaMYB33* may play a role in an antioxidant production to detoxify harmful ROS and may alter the osmotic condition of cells via proline accumulation (Qin et al., 2012). The transcription of *TaPIMP1* MYB was induced by exogenous application of ABA and salicylic acid (SA), suggesting this MYB protein may mediate responses in multiple hormone pathways or serve as an integration point of ABA and SA signaling (Zhang L. et al., 2012). Overexpression of *TaPIMP1* in wheat led to increased drought tolerance and induced expression of the stress-responsive genes *RD22* and *Dehydrin6* (Zhang Z. et al., 2012). *In vitro*, electrophoretic mobility shift assay (EMSA) concluded that TaPIMP1 could interact with multiple MYB-binding motifs (Zhang L. et al., 2012). Some MYB family members, such as TaMYB3R1, appear to play a role in hormone signaling. Exogenous application of ABA and jasmonic acid (JA) were shown to regulate *TaMYB3R1* expression along with osmotic and cold stress (Cai et al., 2011). Interestingly, no downstream targets of TaMYB3R1 were identified, although, it was suggested that R1R2R3-MYB family member might play a role in cell cycle regulation through the interaction of genes with MSA-binding motifs (Cai et al., 2011). TaSIM, an R2R3-MYB TF in wheat was shown to confer salt tolerance when overexpressed in *Arabidopsis* (Yu et al., 2017). Overexpression of *TaSIM* was shown to upregulate expression of *RD22* and *RD29A*, suggesting that this MYB TF played a role in regulating ABA-dependent and independent responses to salinity (Yu et al., 2017). Another recently characterized R2R3-MYB TF plays a positive regulatory role in response to both drought and salt stress (Wei et al., 2017). TaODORANT1 conferred drought and salt tolerance when overexpressed in *Nicotiana tabacum* (Wei et al., 2017). TaODORANT1 plays a role in ABA-mediated stomatal closure and increased production of antioxidant enzymes, superoxide dismutase and catalase under osmotic conditions (Wei et al., 2017).

The function of NAC proteins in ABA-dependent signaling occurs across several species including *Arabidopsis* and rice (Hu et al., 2006; Tran et al., 2007). Improvements to drought and salt stress were accomplished in wheat by expressing a foreign NAC TF, *SNAC1*, isolated from rice (Saad et al., 2013). Increased stress tolerance has been achieved by inducing transcription of genes important to the ABA-regulated pathway. Increased expression of *RCAR*, which is a key component of the ABA receptor likely led to the increased perception of endogenous ABA leading to increased ABA sensitivity and therefore response (Saad et al., 2013). Furthermore, there was increased expression of 1-phosphatidylinositol-3-phosphate 5-kinase (*PI3K*), a key player in ABA-mediated stomatal closure (Saad et al., 2013). The novel NAC TF TaNAC29 induced by salt, PEG, ABA, and H_2_O_2_ when constitutively expressed in *Arabidopsis* led to improved salt and drought tolerance (Huang et al., 2015). Germination assays on ABA demonstrated that *TaNAC29* overexpression conferred ABA-hypersensitivity (Huang et al., 2015). In transgenic *Arabidopsis*, TaNAC29 also increased enzymatic activity of superoxide dismutase and catalase leading to reduced damage by H_2_O_2_ (Huang et al., 2015). Overall, results suggest that TaNAC29 can mediate drought and salt tolerance through ABA-dependent signaling and potential induction of endogenous antioxidant systems. TaNAC47 also plays an important role in response to multiple abiotic stresses (Zhang L. et al., 2016). Transgenic *Arabidopsis*, overexpressing *TaNAC47* had increased tolerance to drought, salt, and cold stress (Zhang L. et al., 2016).

The WRKY-type family of TFs is also believed to play an important role in ABA-dependent abiotic stress signaling. A recent study identified 40 WRKY members in wheat and characterized TaWRKY2 and TaWRK19, both of which were induced by multiple abiotic stresses (Niu et al., 2012). Expression of *TaWRKY2* and *TaWRK19* increased in response to salt, drought and exogenous ABA application. Overexpression of *TaWRKY2* in *Arabidopsis* improved drought and salt tolerance by increasing downstream expression of *STZ* and *RD29B* (Niu et al., 2012). Overexpression of *TaWRKY19* also enhanced salt, drought and cold tolerance (Niu et al., 2012). TaWRKY19 also induced expression of various stress-related genes including *DREB2A, RD29A*, and *RD29B* (Niu et al., 2012). Another WRKY TF, TaWRKY91 is required for alleviating drought stress in an ABA-dependent fashion (Ding et al., 2016). When *TaWRKY91* was overexpressed in *Nicotiana tabacum*, there was increased expression of *NtPYL8* which mediated stomatal closure in an ABA-mediated fashion ultimately leading to improved drought tolerance (Ding et al., 2016).

The bHLH TF family also appears to mediate osmotic stress in an ABA associated fashion. Expression of the wheat bHLH TF *TabHLH1* was shown to increase in response to drought, salt, and exogenous ABA application (Yang T. et al., 2016). Overexpression of *TabHLH1* in tobacco, increased biomass and reduced water loss under osmotic stress conditions (Yang T. et al., 2016). Overexpression of *TabHLH1* led to increased expression of *NtPYL12* and *NtSnRK2.1*, which encode an ABA receptor and kinase respectively, modulating ABA signaling (Yang T. et al., 2016).

Although several downstream genes have been identified, the late embryogenesis abundant/dehydrins (*LEA/DHN)* group of genes play a vital role in preserving cellular components such as membranes (Baker et al., 1988). *DHN-5* was identified in wheat and was transcriptionally activated following salt and ABA treatment (Brini et al., 2007b). Overexpression of *DHN-5* in *Arabidopsis* demonstrated improved tolerance under high salt and water-limiting conditions (Brini et al., 2007a). The endogenous proline levels were higher in *DHN-5* transgenic *Arabidopsis* plants, and the leaves sequestered higher concentration of Na^+^ and K^+^, likely providing better cellular growth conditions (Brini et al., 2007c). Similar to the effects of osmoprotectants such as proline, betaine and glycine betaine, polyamines are shown to reduce ROS damage due to various abiotic stresses (Groppa and Benavides, 2008). The application of exogenous ABA leads to the increase in polyamines such as cadaverine and spermidine (Kovács et al., 2010). Interestingly, *Arabidopsis* mutants deficient in ABA biosynthesis and signaling genes were low in polyamine content (Alcázar et al., 2006) and prone to abiotic stresses, although, it is unclear how ABA mediates polyamines accumulation under stress.

Endogenous ABA levels are strong determinants of the initial responses to various abiotic stresses. The endogenous ABA levels can be manipulated by the application of molybdenum (Mo) (Sun et al., 2009). The effects of Mo on cold stress tolerance have been investigated in winter wheat varieties. Molybdenum is an essential micronutrient that functions as an enzyme cofactor, critical to the function of aldehyde oxidase which converts abscisic aldehyde to bioactive ABA (Kaiser et al., 2005; Sun et al., 2009). Upon application of molybdenum, there was an increase in cold stress tolerance due to an increase in aldehyde oxidase activity, which increased ABA production (Sun et al., 2009). This led to increased expression of downstream bZIP TFs *WLIP19* and *WABI5* and *COR/LEA* genes (*WRAB15, WRAB17, WRAB18, WRAB19)* (Sun et al., 2009). These results suggest that molybdenum is essential for promoting and maintaining biosynthesis of ABA and required to elicit an ABA-dependent stress response (Sun et al., 2009). One potential mechanism for studying influence of ABA in abiotic stress signaling is through utilizing ABA hypersensitive wheat varieties (Kobayashi et al., 2006). Analysis of hypersensitive wheat variety ABA27 demonstrated increased cold tolerance and when ABA27 seedlings were treated with exogenous ABA, displayed enhanced expression of the bZIP TF *WABI5* and other downstream *COR/LEA* (*WDHN13, WRAB15, WRAB18*) genes (Kobayashi et al., 2006). This again suggests the importance of the ABA-dependent expression of *COR/LEA* genes required for cold tolerance. A schematic overview of the ABA-dependent regulation during heat, cold and osmotic stress is presented in **Figure 3**.

The ABA signaling pathway has been the most extensively studied hormonal signaling in wheat. Several homologs of the key players involved in biosynthesis and perception have been identified in wheat (Mao et al., 2010; Zhang et al., 2010; Tian et al., 2013; Tong et al., 2017). Majority of ABA research in wheat has focused on gene expression in response to exogenous ABA application or exposure to abiotic stress conditions. Several families of downstream TFs have been characterized in wheat. Since the core fundamentals of ABA signaling and response have been extensively characterized in the model organism *Arabidopsis thaliana*, there is a reduced body of literature regarding ABA signaling in wheat (see Cutler et al., 2010). The large genome size and ploidy of wheat compared to *Arabidopsis* increases the complexity of understanding hormone signaling pathways in wheat. Future ABA research in wheat will focus on understanding downstream players in ABA signaling allowing breeders to identify genes that could be exploited in a germplasm using marker-assisted breeding or genome editing to confer abiotic stress tolerance.

### Auxin Signaling Is Suppressed During Abiotic Stress Signaling

Auxin, a tryptophan derivative most commonly present in the form of indole-3-acetic acid (IAA), is a critical positive growth regulator involved in numerous aspects of plant growth and development. Auxins primarily regulate and control cell elongation and differentiation (Kosová et al., 2012; Tromas et al., 2013). Auxin-mediated responses are largely the result of transcriptional activation by derepression in which the binding of auxin to its receptor proteins (TIR and AFBs) allows for the targeting and ubiquitination of transcriptional repressors (Aux/IAA repressor proteins) for proteasomal degradation. This allows for the activation of auxin-responsive transcription factors (ARFs) (Qin et al., 2008; Ma X. et al., 2015). Importantly, some auxin receptors and downstream regulated genes have been shown to be regulated by miRNA activity throughout normal development as well as during response to stresses (Tang et al., 2012; Ma X. et al., 2015).

In times of stress, a common method to mitigate the resource strain and overall damage to the plant is to suppress growth to allocate resources to critical processes (Wilkinson et al., 2012). Therefore, under stress conditions, auxin is typically suppressed in its synthesis, signaling, and transport. For example, cold or frost stressed wheat plants have a decreased accumulation of auxin in the leaves, either through reduced biosynthesis or impeded PIN protein trafficking (Kosová et al., 2012). Furthermore, in a frost-susceptible variety, maintaining a higher level of auxin following an acclimatization period was associated with lower fitness (Kosová et al., 2012). Wheat plants under drought and salt stress during reproductive growth exhibited reduced grain filling which was associated with lower levels of auxin (Abid et al., 2017). Furthermore, expression of auxin receptors and transporters was observed to be downregulated in the roots of drought exposed wheat plants in both susceptible and resistant genotypes (Krugman et al., 2011). In *Arabidopsis*, salt stress has been shown to stabilize some AUX/IAA repressor proteins as well as downregulate the expression of PIN proteins in the roots, which together result in reduced auxin signaling and inhibited root elongation (Liu W. et al., 2015). In reproductive organs, notably, the anthers, heat stress can disrupt and inhibit auxin biosynthesis and is associated with heightened pollen abortion and male sterility (Sakata et al., 2010). Furthermore, application of auxin to the developing anthers during heat stress was sufficient to rescue the male-fertility (Sakata et al., 2010). From a biotechnological standpoint, being so prevalent and critical throughout development, constitutive or unregulated alterations to auxin synthesis, signaling, or transport tend to have negative pleiotropic effects. However, sensitive tissues such as anthers and developing seeds or grains could be designed to maintain sufficient auxin accumulation through the use of tissue-specific and stress-inducible promoters driving the transcription of auxin biosynthetic genes. This could allow sustained reproduction in spite of challenging environmental conditions. Studies of auxin interactions in wheat have primarily focused on auxin abundance under varying conditions or the effects of exogenous application during times of stress. Genomics studies have thus far heavily relied upon the gene annotations of model organisms (i.e., rice and *Arabidopsis*) for the identification of homologous genes. This allows for the identification of inferred functions and responses, often based on gene expression level. Further characterization of these identified auxin-responsive or regulatory genes is needed for tailoring of auxin signaling in wheat plants for optimal growth and development under a variety of environmental stresses.

### Targeted Suppression of Cytokinin Responses Could Confer Stress Tolerance Without Affecting Grain Filling

Cytokinins are another group of positive growth regulators that function throughout development; however, they act contrastingly with auxin. Cytokinins primarily promote cell division and proliferation and are key regulators of the shoot apical meristem in that they help maintain a population of repeatedly dividing, undifferentiated cells (Su et al., 2011). The induction of cellular division by cytokinins is critical for the early stages of embryogenesis as well as endosperm proliferation for the production of full grains (Abid et al., 2017). Cytokinins also play an important role in the prevention of cellular damage by ROSs, in particular, aiding in the retention of chlorophylls during stress, shade, or dark-induced senescence of the leaves (Zavaleta-Mancera et al., 2007; Wilkinson et al., 2012). Cytokinins have been shown to reduce the activity of chlorophyllases and dechelatases (which act to break down chlorophyll during senescence), as well as increasing the activity of antioxidant enzymes (such as catalase and ascorbate peroxidase) and the accumulation of the antioxidant molecules, xanthophylls (Zavaleta-Mancera et al., 2007). Active cytokinins are perceived extracellularly at the plasma membrane or internally at the ER by membrane-bound receptor proteins known as Histidine-kinases (HKs) which will then undergo autophosphorylation and begin the signal transduction *via* transfer of the phosphoryl group to a His-phosphotransfer protein (HP) which will subsequently localize to the nucleus to phosphorylate a response regulator (RR) (Osakabe et al., 2013; Sun et al., 2014). Importantly, the signaling of cytokinin has been found to be antagonistic to the signaling of abscisic acid (ABA) (and vice versa), which is critical when assessing plant responses to abiotic stresses (Wilkinson et al., 2012). Another key and a well-studied regulatory component of cytokinin signaling is the enzyme family responsible for cytokinin deactivation, cytokinin oxidase/dehydrogenases (CKXs) which can be positively regulated by ABA (Zalewski et al., 2010; Kosová et al., 2012; Sun et al., 2014). As in the case of auxin, the majority of plant tissues will decrease cytokinin production and signaling during times of stress to limit strain as well as to enhance the plant’s sensitivity to ABA for mediation of stress responses (Zalewski et al., 2010; Kosová et al., 2012; Sun et al., 2014; Abid et al., 2017). In cold-stressed wheat plants, cytokinin levels are suppressed early in a cold-tolerant cultivar whereas the cold-susceptible cultivar suppressed cytokinin later and not to the same extent which was correlated with reduced plant fitness (Kosová et al., 2012). Under drought conditions, it has been shown that the suppression of cytokinin biosynthetic enzymes (i.e., IPT), as well as suppression of positive cytokinin signaling components (HKs) can lead to improved drought-tolerance (Nishiyama et al., 2011; Sun et al., 2014). Alternatively, studies done in tobacco have shown that senescence- and maturation-inducible expression of an IPT enzyme allowed for heightened drought tolerance due to the maintenance of photosynthetic capacity under water deficit (Rivero et al., 2007, 2010). This was accompanied by suppression of ABA-mediated responses and reduced senescence of the mature leaves during the drought treatments. Although this may be beneficial under mild drought stress in wheat, failure to senesce in the lower leaves under severe drought could reduce the nutrient assimilation required for proper grain development and the suppression of ABA-responses would enhance drought susceptibility (Nishiyama et al., 2011). There are, however, regions of the plant that could still benefit from cytokinin production during stress, in particular developing grains (Wilkinson et al., 2012; Abid et al., 2017). By inducing the proliferation of the endosperm, the plants obtain a greater “sink” in the developing grains which will aid in proper grain filling and maturation while under stress. As the reproductive stages are so sensitive to stress, drought exposed wheat plants may display impaired grain filling which is associated with reduced auxin and cytokinin levels but can be partially rescued by exogenous application of cytokinin (Abid et al., 2017). As in the case of auxin, large-scale disruption of cytokinin synthesis and signaling will likely result in negative pleiotropic effects, however, targeted tissue expression of cytokinin biosynthesis enzymes or the silencing of cytokinin signaling components may be helpful innovations for improving grain yield and maintaining drought tolerance simultaneously. Many of the studies analyzing cytokinin interactions in wheat and model organisms have focused on the effects of modifications to cytokinin biosynthesis or degradation and the quantification of active cytokinins through development and times of stress, often completed as comparative studies between stress-susceptible and resistant varieties. A majority of the studies focusing on the characterization of signaling components have involved model plants (i.e., rice, *Arabidopsis*, and tobacco) or closely related cereals such as barley. Further identification and potential manipulation of cytokinin-responsive genes is critical for developing a firmer understanding of the effects of cytokinin on wheat development and responses to stress.

### Modulation of Gibberellic Acid Can Promote Abiotic Stress Tolerance

Gibberellins (GA) represent a large group of plant hormones characterized as tetracyclic diterpenoid carboxylic acids. They act as key regulators of growth throughout various stages of plant development via regulation of cell elongation and division. The biosynthesis of these compounds involves the action of various GA dioxygenases, while a key dioxygenase (GA2ox) is involved in the homeostatic control of GA levels through its action in catalyzing the production of inactive GA (Colebrook et al., 2014). GA signaling involves the targeted degradation of a group of GA-response transcriptional repressors, DELLA proteins, upon GA perception (Golldack et al., 2014). This signaling pathway was shown to be paramount to success of wheat seedlings in the Mediterranean soil where the uppermost section of soil is arid and water deficient (Amram et al., 2015). GA insensitive *Rht* mutants exhibit a dwarf phenotype which reduces their successful emergence under deep sowing conditions important under these soil conditions (Amram et al., 2015). Stressful environmental conditions are known to restrict plant growth through the modulation of endogenous GA levels as mutant lines with reduced GA content exhibit a salt-tolerant phenotype (Achard, 2006). This is also confirmed by the observation that endogenous GA levels are suppressed under drought and salt stress either through a reduction in biosynthesis or in elevation of degradation (Llanes et al., 2016). Using GA-inhibiting compounds, it has been shown that crop stress-tolerance under drought conditions can be achieved while at the same time increasing biomass and yield (Plaza-Wüthrich et al., 2016). Several important crop species, such as rice and barley, are known for their dwarf cultivars. These varieties have been shown to exhibit stress tolerant phenotypes due to their reduced GA levels and these characteristics were abolished following exogenous treatment of these varieties with GA (Vettakkorumakankav et al., 1999). A biotechnological application of this pathway to engineering superior crop varieties may simply focus on the *GA2ox* genes. A complete reduction of GA will inevitably lead to detrimental effects on crops, therefore, targeting this group of enzymes involved in homeostatic control of GA may prove to be a more successful strategy. This was shown through the ectopic expression of several GA2ox genes in rice, whereby the moderate reduction in GA resulted in lodging resistant, dwarf plants with higher water use efficiency, increased abiotic stress tolerance, and an increase in productive tiller count (Lo et al., 2017). These studies demonstrate the complexities and trade-offs involved in single-pathway manipulations, stressing the need for more intricate multi-pathway approaches to crop-improvement strategies. Although there has been some investigation into specific GA sensitivity genes in dwarfed wheat varieties in connection to yield and emergence (Haque et al., 2011), the majority of work elucidating the role of GA in development and stress has come from other organisms. Future work should focus on GA’s role in yield and its potential crosstalk with other hormones involved in yield and stress tolerance.

### Exogenous Application of Brassinosteroid Can Improve Abiotic Stress Tolerance

Brassinosteroid is a steroid plant hormone which plays an important role in plant growth, development and defense responses. Being ubiquitously present throughout the plant brassinosteroids impact a wide range of regulatory functions such as cell elongation, cell division, vascular differentiation, biotic and abiotic responses, and senescence (Clouse and Sasse, 1998). The effect of brassinosteroid compounds (BRs) has been investigated on various aspects of wheat growth and development during the last three decades but not much has been explored at the molecular level. In earlier studies, the effect of foliar BR application was investigated on wheat seedlings; application of BR resulted in overall growth improvement, particularly enhanced photosynthate accumulation, higher CO_2_ fixation and RubPCase activity, which augmented fresh and dry weight of leaves and shoots (Braun and Wild, 1984). When the influence of exogenously applied active BR compound, 2,4-epibrassinolide (EB) was examined based on physiological effects such as chemical composition of grains and yield, EB increased grain yield by 20%, but no change in chemical composition was found (Janeczko et al., 2010). However, in cultivated wheat, soluble sugar contents were found to be higher than control plants, while total fats and calcium were lowered with no change in overall starch and protein content (Janeczko et al., 2010). BRs are also known to interact with other hormones to influence abiotic stress tolerance. Preconditioning with EB reduced the levels of stress-induced ABA and IAA, while cytokinin concentration remained unaltered (Avalbaev et al., 2010). Similar results were also found in another study where the drought was induced with 5% mannitol and seeds were treated with 0.4 μM 24-epibrassinolide (EB) before sowing (Shakirova et al., 2016). However, in a different study, application of 0.4 μM 24-epibrassinolide to growing seedlings of wheat (*Triticum aestivum* L.) showed enhanced cytokinin levels in roots and shoots of 4-days grown seedlings but these levels were only maintained in the presence of EB, whereas the removal of hormone led to return of normal cytokinin levels as found in control plants (Yuldashev et al., 2012). This EB-induced increase in cytokinin level was concomitant with the inhibition of cytokinin oxidase (CKX) activity and reduced the expression of the *CKX* gene (Yuldashev et al., 2012). The impact of BRs as a protective agent was also examined under lead, cadmium (Cd) and salinity (NaCl) stress on *Triticum aestivum*. The presence of cadmium and salinity normally reduced the plant growth and photosynthetic activity but enhanced antioxidant enzyme activity and proline content (Hayat et al., 2014). Spraying with 28-homobrassinolide (HBL), eliminated these detrimental effects of Cd/NaCl and further improved antioxidant enzyme activity and proline contents (Hayat et al., 2014; Tofighi et al., 2017). BRs not only improved plant growth status under heavy metal stress but also lowered the contents of heavy metals (Cu, Cd, Pb, and Zn) when wheat plants were grown on soil contaminated with these heavy metals (Kroutil et al., 2010).

In spite of extensive research on the influence of exogenous application of BR, only a few reports have shown the manipulation of BR receptors and its downstream targets to examine its impacts on signaling and growth parameters in wheat. During canonical BR signaling, a transmembrane protein receptor kinase, brassinosteroid-Insensitive 1 (BRI1) interacts with BR to control plant growth (Navarro et al., 2015). A detailed analysis of the BR receptor and other signaling components have been done in *Arabidopsis*, but there is only limited knowledge of BRI1 orthologs available in other taxa. *In silico* analysis of three wheat genomes have shown the presence of a single copy of *BRI1* (*TaBRI1*) in each genome on the long arm of chromosome 3 (Navarro et al., 2015). Like *Arabidopsis* BRI1, TaBRI1 possesses multiple leucine-rich repeats (LRRs), an island domain (ID), a juxtamembrane/transmembrane domain (JTMD), a catalytic kinase domain (KD), with C and N-terminal domains similar to ATBRI1 (Navarro et al., 2015). Later on, functional analysis of TaBRI1 was done in *Arabidopsis thaliana* through transgenic overexpression which revealed interaction of TaBRI1 with five members of wheat Somatic Embryogenesis Receptor Kinase family (TaSERK1, TaSERK2, TaSERK3, TaSERK4, and TaSERK5) at the plasma membrane (Singh et al., 2016). Furthermore, TaBRI1 overexpression in *Arabidopsis* plants showed increased thermotolerance, hypersensitivity to epi-brassinolide in roots, early flowering, larger siliques and improved yield, mimicking the BR signaling responses found in various studies in *Arabidopsis* shown earlier (Singh et al., 2016). Downstream of BRI1, the glycogen synthase kinase, BIN2, functions as a negative regulator of BR signaling and has been vastly investigated in *Arabidopsis* and two homologs of wheat GSKs, TaSK1 and TaSK2 were studied in *Arabidopsis* and *Triticum aestivum* to dissect their role in BR signaling (Bittner et al., 2015). When homologous copies of TaSK1 and TaSK2 were mutated similar to a *bin2-1* gain of function mutation and expressed in Arabidopsis, it phenocopied *bin2-1* with severe dwarfism and down-regulation of downstream BR genes *SAUR-AC1, CPD* and *BAS1* (Bittner et al., 2015). Treatment with bikinin (GSK inhibitor) partially rescued this dwarf phenotype and altered the expression of these genes (Bittner et al., 2015). Taken together, although *in vivo* genetic data is lacking to directly prove that BR can improve abiotic stress tolerance, in general, exogenous application of physiologically high levels of BR, seems to significantly improve abiotic stress tolerance in wheat.

### Strigolactone Manipulation Could Provide New Avenues for Generating Stress Tolerant Wheat Germplasm

Strigolactones (SL) are a group of plant hormones involved in developmental programs as well as symbiotic relations in the rhizosphere (Cheng et al., 2013). They are carotenoid-derived terpenoid lactone class of molecules with a tricyclic ABC ring covalently bound to a D ring through an enol-ether bridge (Waldie et al., 2014). This enol-ether bridge is thought to be the bioactiphore of all SL-family molecules (Lopez-Obando et al., 2015). First identified as a germination stimulant of parasitic weed species *Striga* and *Orobanchaceae*, it was later associated with shoot and root architectural regulation with its ability to suppress axillary meristem activity (Janssen et al., 2014). Aside from its role in development, it has also been linked to abiotic stress response in plants. Although very little work has been done on SL and stress in wheat, studies from other species may shed light on conserved mechanisms (Liu J. et al., 2015).

As SLs are primarily synthesized in the roots (Al-Babili and Bouwmeester, 2015), it would be natural for them to take part in abiotic stress responses associated with such tissues. The downregulation of an SPL gene (*SPL8*) in alfalfa resulted in an increased branching phenotype (characteristic of SL-related mutants), but more importantly led to increased resilience against salt and drought stress in these lines (Gou et al., 2017). The application of a synthetic SL analog (GR24) to *Brassica napus* improved the growth of these plants under high-salt conditions as well as increased the activities of antioxidant enzymes superoxide dismutase and peroxidase (Ma N. et al., 2017). As SL is transported acropetally from the roots to the shoots (Brewer et al., 2015), it may be a good candidate for a systemic signal for root-perceived stresses. In tomato, drought stress repressed SL biosynthesis gene activity in roots while increasing the activity of these same genes in the shoots (Visentin et al., 2016). This may be a result of the feedback regulation under which these genes are controlled whereby a decrease in SL signal from the roots may induce upregulation of biosynthesis in shoots. SL-depleted plants exhibited hypersensitivity to drought likely caused by a reduction in ABA-induced stomatal closure (Visentin et al., 2016) causing higher transpiration rates (Liu J. et al., 2015). Based on this observation, it can be concluded that SL plays a crucial role in abiotic stress responses in close association with ABA. Drought stress was shown to reduce thousand grain weight as well as yield in two winter wheat cultivars *(Triticum aestivum L.)*, this being significantly alleviated when plants were exogenously treated with synthetic SL analog GR24 (Sedaghat et al., 2017). This hormone may hold the key to biotechnological advancements in the agricultural protection of adverse climatic conditions, specifically salt and drought, simply through its ability to enhance drought tolerance via exogenous application (Ha et al., 2014).

An agronomically important abiotic stress, one which is seldom considered, is high wind and rain. High-velocity wind/rain may result in the lodging of important crops resulting in loss of yield. As water deficiency may not be a problem in areas with such conditions, the drought would be of lesser concern than breakage and lodging of the stem. This is important in considering the wide variety of applications surrounding the manipulation of the SL pathway. As was previously mentioned, exogenous SL application may assist in drought resistance to plants in those parts of the world where water is of major concern, but the SL-deficient dwarf mutants may prove useful in extreme weather conditions where lodging is problematic. Aside from the high-branching phenotypes characteristic of SL-deficient or insensitive mutants, lack of SL also exhibits a dwarf phenotype (Bennett et al., 2016) which may mitigate yield losses resulting from crop lodging (Berry et al., 2004). SL is known to be a regulator of shoot and root architecture, specifically in relation to soil nutrient quality (Mishra et al., 2017). However, our knowledge of SL’s connection to abiotic stress is scarce, even in the model organisms. SL research in wheat, specifically, is highly unexplored relative to rice and *Arabidopsis*. As there is evidence of species-specific variation in SL function which is linked to the developmentally regulated physical size of the organism of study (Foster et al., 2017), future research should aim to elucidate the wheat-specific regulatory networks underlining SL’s role in abiotic stress.

### Salicylic Acid Application Can Mitigate Harmful Effects of Abiotic Stresses

Salicylic acid is an endogenous plant hormone that regulates various processes including: growth and development, germination, photosynthesis, and antioxidant regulation (Singh and Usha, 2003; Afzal et al., 2005; Arfan et al., 2007; Khan et al., 2013; Janda and Ruelland, 2015; Chen et al., 2016; Noreen et al., 2017; Fardus et al., 2018). The majority of research involving SA has primarily focused on its role when exogenously applied under various abiotic stresses (see below).

Exogenous application of SA in wheat has demonstrated the ability of SA to act as a signaling molecule to induce an endogenous radical detoxification system (Singh and Usha, 2003; Agarwal et al., 2005a,b; Arfan et al., 2007; Khan et al., 2013; Chen et al., 2016; Noreen et al., 2017; Fardus et al., 2018). Exogenous application of SA in wheat led to an increased production of antioxidants, leading to reduced instances of oxidative damage under heat, drought, and saline conditions (Singh and Usha, 2003; Agarwal et al., 2005a,b; Arfan et al., 2007; Khan et al., 2013; Chen et al., 2016; Noreen et al., 2017; Fardus et al., 2018). SA can activate antioxidant production using ROS as a second messenger (Agarwal et al., 2005a,b). Exogenous application of SA were used to mitigate damage caused by abiotic stresses by increasing the production of H_2_O_2_ in an NADPH-oxidase-dependent manner (Agarwal et al., 2005a,b). The increased production of H_2_O_2_ and ROS stimulate the production of antioxidants to detoxify the accumulating ROS (Agarwal et al., 2005a,b). Exogenous SA applications of 1.0 mM in wheat led to increases of superoxide dismutase, ascorbate peroxidase, glutathione reductase, and catalase (Singh and Usha, 2003; Agarwal et al., 2005a,b; Chen et al., 2016). Lower concentrations of SA generally were more effective because SA application slightly increased hydrogen peroxide production through NADPH-oxidase (Agarwal et al., 2005a,b). Treatment of wheat seedlings with 0.5 mM of SA helped alleviate growth inhibition that can be induced by drought stress (Agarwal et al., 2005a,b; Noreen et al., 2017). The application of SA in wheat increased the production of two key antioxidants glutathione and ascorbate. Furthermore, it was identified that multiple genes encoding enzymes within the ascorbate-glutathione cycle were upregulated (Kang et al., 2013). It is also important to note that high concentration applications (2.5 mM) of exogenous SA under abiotic stress can promote oxidative damage due to accumulation of O_2_^⋅-^, H_2_O_2_, and malondialdehyde (MDA) (Chen et al., 2016). In addition to accumulation of ROS, there was evidence of membrane damage due to indication of extensive electrolyte leakage (Chen et al., 2016). This suggests the potential antagonistic effect when applying exogenous SA in large quantities.

The exogenous application of SA in wheat also appears to improve photosynthetic capacity under abiotic stress (Singh and Usha, 2003; Arfan et al., 2007; Khan et al., 2013; Chen et al., 2016). The accumulation of oxidative radicals can inflict damage on membranes, ultimately impacting processes including photosynthesis (Chen et al., 2016). Increasing antioxidant production would reduce oxidative damage to photosynthetic membranes, preserving photosynthetic processes upon SA application (Chen et al., 2016). The application of SA likely protects photosystem II by regulating the abundance of various thylakoid membrane proteins (Chen et al., 2016). In wheat, under heat stress exogenous application of 0.5 mM SA led to increased accumulation of proline, which promoted osmotic and water relations to preserve photosynthetic activity (Khan et al., 2013). Exogenous SA application also decreased the production of stress-related ethylene by inhibiting ACC synthase in wheat (Khan et al., 2013). This led to more favorable ethylene levels promoting improved photosynthesis under heat stress conditions (Khan et al., 2013). This result suggests an antagonistic effect between SA and ethylene but needs further investigation to understand the signaling mechanism. Exogenous SA application also appears to help preserve photosynthetic function in drought stressed wheat crops by promoting increased antioxidant production (Arfan et al., 2007). SA application on wheat plants under drought also appears to have a positive effect on RUBISCO activity and maintaining chlorophyll levels, resulting in increased growth response (Singh and Usha, 2003).

The field of SA research in wheat has largely revolved around the exogenous application of SA and the corresponding cellular responses. It is evident that the application of exogenous SA in wheat can activate an endogenous radical detoxification system to eliminate potentially harmful oxygen species (Singh and Usha, 2003; Agarwal et al., 2005a,b; Arfan et al., 2007; Khan et al., 2013; Chen et al., 2016; Noreen et al., 2017; Fardus et al., 2018). The signaling mechanism behind SA response to abiotic stress has not been extensively investigated in wheat, compared to other model systems (Horváth et al., 2007). It is likely that SA signaling may be complex due to the antagonistic nature of SA when applied in high concentrations (Chen et al., 2016). Future SA research will continue looking at gene expression following exogenous SA application in attempts to understand how SA signaling could be utilized to mitigate oxidative damage induced by abiotic stress.

### Abiotic Stresses Are Integrated Through Jasmonic Acid Signaling

The role of JA has primarily been investigated in response to biotic stress and pathogen attacks. However, there is an increasing amount of research that has been investigating its role in response to abiotic stress. The majority of research conducted thus far has looked at exogenous applications of methyl jasmonate (MeJA) or JA and its ability to confer abiotic stress tolerance. The role of JA in abiotic signal transduction is not entirely understood, but advancement in the field will allow for new strategies to combat abiotic stress.

Exogenous application of 2 mM JA in wheat seedlings led to an increase in salt tolerance. This study focused on the role of JA and its link to the production of antioxidants which can mitigate the damage caused by ROS accumulation after exposure to stress (Qiu et al., 2014). Following JA application there was increased expression and enzymatic activity of superoxide dismutase, peroxidase, catalase, and ascorbate peroxidase (Qiu et al., 2014). This led to a reduction of H_2_O_2_ and other ROS, which would otherwise be harmful to cellular survival. Like SA treatment, there is evidence suggesting that exogenous JA application can alleviate drought stress by regulating the ascorbate-glutathione pathway (Kang et al., 2013; Shan et al., 2015). Application of JA to wheat seedlings followed by drought stress, induced activities of AsA-GSH players (APX, GR, MDHAR, DHAR) which led to increased tolerance under drought conditions, likely through reducing oxidative damage induced by ROS. There was also reason to believe the induction of the AsA-GSH pathways was mediated by nitric oxide (NO) following exogenous JA application (Shan et al., 2015). When JA treated wheat seedlings under drought stress were exposed to a NO inhibitor, the improved drought tolerance was abolished suggesting the importance of NO transducing the signal from JA to the AsA-GSH cycle (Shan et al., 2015).

Jasmonic acid signaling may play an important role bridging abiotic and biotic stress tolerance. The TaMYB33 TF (discussed in ABA section) may also play a role in the JA signaling pathway. The promoter sequence of *TaMYB33* contains four potential MeJA-responsive motifs indicating its potential role in JA hormone signaling (Qin et al., 2012). When *TaMYB33* was overexpressed in *Arabidopsis*, there was upregulation of a known biotic defense gene (*PDF1.2a*) suggesting *TaMYB33* could be a link integrating both biotic and abiotic stress (Fujita et al., 2006; Qin et al., 2012). The characterization of a novel wheat expansin gene *TaEXPB23* has also opened new avenues for the role of jasmonate-mediated abiotic stress tolerance. The expression of *TaEXPB23* was induced under drought stress and following exogenous MeJA application (Han et al., 2012). The promoter region of *TaEXPB23* contained a promoter motif that is responsive to MeJA suggesting that MeJA can mediate expression to some capacity (Han et al., 2012). Overexpression of *TaEXPB23* in tobacco increased tolerance under salt stress, however, the function of TaEXPB23 has not been characterized. The characterization of a wheat allene oxidase cyclase, TaAOC1, has also provided insight on the link between jasmonate-mediate signaling and abiotic stress tolerance (Zhao et al., 2014). In wheat seedlings, expression of *TaAOC1* was induced by NaCl, PEG, and hormone applications of JA and ABA (Zhao et al., 2014). When *TaAOC1* was overexpressed in both wheat and *Arabidopsis*, there was an increase in salt tolerance. Further investigation in *Arabidopsis* overexpression lines suggests that overexpression of *TaAOC1* increased JA biosynthesis and accumulation of *MYC2* transcript (Zhao et al., 2014). When *TaAOC1* was overexpressed in the *myc2-2* mutant, the enhanced salt tolerance diminished suggesting MYC2 is an important downstream player in mediating a JA-dependent signal (Zhao et al., 2014). It appears that MYC2 appears to be a converging point in ABA and JA signal transduction in response to salt stress. (Dong et al., 2013; Zhao et al., 2014).

The JA signaling pathway has been extensively characterized in *Arabidopsis*, with a large focus on response to biotic stress (Turner et al., 2002). An increasing body of research suggests that JA signaling also plays an important role in the response to various abiotic stresses (Ahmad et al., 2016). The limited JA signaling research in wheat, suggests that JA plays an important role in the induction of an antioxidant system that helps remove potentially harmful ROSs (Kang et al., 2013; Qiu et al., 2014; Shan et al., 2015). There is also evidence that JA and ABA may have a convergence point which is integral for abiotic stress responses (Dong et al., 2013; Zhao et al., 2014). Future research in the field of JA hormone signaling in wheat will explore the link between biotic and abiotic stresses, and how we may be able to mitigate damage due to multiple stresses by exploiting the same signaling pathway.

### Ethylene Inhibitors Promote Abiotic Stress Tolerance

Ethylene is a gaseous hormone generated from *S*-adenosylmethionine (SAM, the same precursor for polyamines) and largely acts as a negative growth regulator (Kosová et al., 2012; Gharbi et al., 2017; Yang W. et al., 2017). Ethylene is primarily involved in the late stages of plant growth and development, particularly senescence and associated leaf abscission, and fruit ripening in preparation for seed maturation, drying, and dispersal (Hays et al., 2007; Xu et al., 2007; Wilkinson et al., 2012). Ethylene also acts throughout development to halt or redirect growth and nutrient allocation in response to numerous environmental cues and stresses including heat, salt, and drought stresses, as well as shading, flooding, pollution, and soil compaction (Wilkinson et al., 2012; Gharbi et al., 2017; Vysotskaya et al., 2017; Yang W. et al., 2017). Ethylene release has been associated with the inhibition of root growth and leaf expansion, and, if induced by severe or extended stresses, can promote extensive plant senescence, embryo abortion, and reduce grain filling (Hays et al., 2007; Wilkinson et al., 2012). Ethylene, being gaseous, can be difficult to detect and, after release, readily diffuses into the surrounding air. As such, many analyses focus on the production and accumulation of ACC as a proxy for ethylene biosynthesis as it has been found that ACC oxidase activity is largely constitutive throughout plant tissues and so ACC levels can be accurately related to levels of ethylene production (Habben et al., 2014; Yang W. et al., 2017).

Ethylene, like cytokinin, is perceived at the cell membrane or ER by membrane-bound HKs which undergo autophosphorylation to begin the Histidine-Aspartate phospho-relay system through phosphate transfer by His-phosphotransfer proteins which will then activate TFs known as the ERFs (Ethylene Responsive Factors) (Xu et al., 2007; Osakabe et al., 2013; Rong et al., 2014). Interestingly, ethylene acts antagonistically toward ABA by reducing its accumulation (and vice versa) which can be beneficial for the mediation of stress responses induced by mild stressors, aiding in temporarily desensitizing plant tissues to ABA and allowing for continuation of normal metabolism (i.e., maintains stomatal conductance and photosynthesis) (Xu et al., 2007; Wilkinson et al., 2012). On the contrary, activation, and expression of some ERF proteins, including TaERF1 and TaERF3 have been shown to mitigate tolerance to multiple stresses, can be induced by either ABA or ethylene perception (Xu et al., 2007; Rong et al., 2014). As suggested earlier, the severity and duration of the stress will dictate the extent of ethylene evolution and therefore the level of response. Under severe drought stress, *ACS* (ACC synthase) is highly upregulated which allows for ACC accumulation to greatly increase in developing grains which has a strong inhibitory effect on the enzymes required for starch synthesis and accumulation (Sucrose Synthase, ADP-Glucose Phosphorylase, and Soluble Starch Synthase) and is therefore associated with reduced grain filling. These effects were significantly reduced with the application of the ACS inhibitor, aminoethoxyvinylglycine (AVG) (Yang W. et al., 2017). Heat stress produces similar effects on ACC accumulation as drought and has been shown to induce kernel abortion, reduce grain filling, hasten seed maturation and desiccation, and initiate early-onset senescence of flag leaves. However, following treatments with the ethylene receptor inhibitor, 1-methylcyclopropane (1-MCP), all of the above symptoms were remediated in the same cultivar under the same heat stress (Hays et al., 2007). The effect of ethylene on salt stress tolerance is still unclear, however, it has been shown that some ERFs play an important role in mediating the salt stress response, and heightened expression of these factors has been associated with maintained photosynthetic capabilities, enhanced osmolyte accumulation, and reduced H_2_O_2_ accumulation, enhancing tolerance to salt, drought, and cold stresses (Xu et al., 2007; Rong et al., 2014). Similarly, the application of ethylene synthesis inhibitors in other species, such as tomato (*Solanum* spp.), has been shown to greatly reduce salt tolerance (Gharbi et al., 2017). Application of ethylene perception and/or biosynthesis inhibitors during periods of severe drought or heat stresses may aid in the reduction of stress-induced ethylene responses, allowing for the maintenance of regular growth and improved yield. Additionally, upregulation or overexpression of some *ERF* genes (e.g., *TaERF1* and *TaERF3*) may enhance overall tolerance to salt, drought, and cold stresses (Xu et al., 2007; Rong et al., 2014). Furthermore, as polyamines (such as putrescine, spermine, and spermidine) share the same precursor as ethylene, enhancing their production has been shown to improve plant stress responses under drought and salt stresses, and could limit the availability of SAM for ethylene production under times of stress. Although some studies have provided valuable information on ethylene-responsive factors through gene characterization in wheat, a majority of studies have focused on the modification of ethylene production or the abundance of ethylene (or its precursor, ACC) under varying environmental conditions. Further analysis of ethylene signaling components may prove critical for the specified modification of ethylene responses under stressful environmental conditions.

## Future Perspectives

Global food security is a major concern being addressed worldwide. Rapid population increases and unpredictable climatic events continue to pressure the need to increase crop productivity. Climatic events can induce abiotic stresses not limited to drought and temperature stress, while diminishing soil conditions can enhance saline stress. Wheat is an important crop accounting for a large portion of human caloric consumption, therefore minimizing abiotic stress is critical to preserve global food security. Understanding perception and signaling cascades activated in response to abiotic stress will provide valuable information which can be used to design new technologies to mitigate yield loss induced by abiotic stress. Identifying novel players involved in abiotic stress signaling will allow researchers to design new technologies and farming strategies for mitigating abiotic stress. This review has focused on advancements of abiotic stress signaling in wheat, particularly involved in hormone-mediated signaling pathways. The key would be to generate new wheat germplasms that can maintain their growth vigor and yield traits while maintaining an enhanced ability to withstand adverse environmental assaults. It is possible to design such strategies to mitigate abiotic stress by exploiting hormone-dependent signaling. Conventional breeding programs can use information regarding hormone signaling networks to breed lines with altered expression levels of important regulatory players mediating stress responses. Plant hormones also have the potential to be utilized as a pre-seeding treatment or as an in-season foliar application. There also appears to be potential for generating transgenic wheat varieties that have altered expression of upstream regulators or downstream stress responsive genes involved in various abiotic stresses. This, however, seems like an unlikely avenue due to the regulatory agencies and public fear of genetically modified organisms. Alternatively, the identification of negative regulatory elements involved in hormonally linked abiotic stress signaling pathways may also prove valuable with the advancement of CRISPR/Cas9 based genome editing technology available in plant systems. Ultimately, the advancement and identification of novel stress signaling players will be critical for designing technologies to increase plant productivity and yield when exposed to unfavorable growth conditions.

## Author Contributions

MSa, KA, and LS conceived the idea and came up with the topics. KA wrote majority of the general perception and signaling during abiotic stress. LS wrote the ABA section and helped with tying-up the whole review. MSt, NH, and MJ wrote sections on hormonal interactions during abiotic stress signaling.

## Conflict of Interest Statement

The authors declare that the research was conducted in the absence of any commercial or financial relationships that could be construed as a potential conflict of interest.

## Footnotes

1 https://www.ipcc.ch/index.htm
